# *In vitro* and *in vivo* inhibitory effects of *Carica papaya* seed on α-amylase and α-glucosidase enzymes

**DOI:** 10.1016/j.heliyon.2020.e03618

**Published:** 2020-03-25

**Authors:** Reuben Agada, Wurochekke Abdullahi Usman, Sarkiyayi Shehu, Dluya Thagariki

**Affiliations:** a Department of Biochemistry, School of Life Sciences, Modibbo Adama University of Technology Yola, Adamawa State, Nigeria; b Department of Biochemistry, Adamawa State University Mubi, Nigeria

**Keywords:** Type 2 diabetes, *Carica papaya*, Seeds extracts, Phytochemicals, Enzyme inhibitors, amylase, glucosidase, Hyperglycemia, Bio-active comounds, GC-MS analysis, Chemistry, Food science, Environmental science, Biological sciences, Health sciences

## Abstract

The present study was aimed to investigate the *in vitro* and *in vivo* inhibitory effects of *Carica papaya* seeds on α-amylase and α-glucosidase enzymes, as this is known to be an antidiabetic mechanism. Analysis of the extracts of the seeds for phytochemicals revealed the presence of a significant amount of saponins, alkaloids, flavonoids, phenols, terpenoids, and steroids. The seed extracts of *Carica papaya* exhibited good antioxidant capacity using 2, 2-diphenyl-1-picryl hydroxyl (DPPH), thiobarbituric reactive substance (TBARS) and ferric reducing antioxidant power (FRAP) method. The results of the inhibitory studies of the extracts revealed that the hexane extract followed by the ethyl acetate extract was the most potent inhibitor of α-amylase and α-glucosidase enzyme when compared to other extracts using their IC_50_ values. In the animal study, different doses (250, 500 and 1000 mg/kg/body weight) of the extracts of *Carica papaya* seed were administered orally for 120 min, to normal and streptozotocin-induced diabetic rats, and were compared with acarbose 100 mg/kg/body weight and control group for the effect on postprandial hyperglycemia. The extract of ethyl acetate (at doses of 250, 500 and 1000 mg/kg/body weight) significantly reduced postprandial glucose levels in these animals. The characterization of hexane and ethyl acetate extracts by GC-MS analysis revealed 20 bioactive compounds while the FTIR analysis confirmed the presence of this functional groups: -C=C, -C-Cl, -C-O, -O-H, -CH, -C=O, -C=C=C, -N=C=S, -O=C=O and -N-H in *Carica papaya* seed extracts. It was concluded that the inhibition of α-amylase and α-glucosidase enzymes and the prevention of oxidative stress in postprandial hyperglycemia could be some of the possible mechanisms by which they exert their anti-diabetic properties.

## Teaser

Type 2 diabetes; *Carica papaya*; Seeds extracts; Phytochemicals; Enzyme inhibitors; <alpha>-amylase; <alpha>-glucosidase; Hyperglycemia; Bio-active comounds; GC-MS analysis; Chemistry; Food science; Environmental science; Biological sciences; Health sciences

## 1 Introduction

Diabetes Mellitus (DM) is a progressive metabolic disease that has negatively affected human health and overall wellbeing. It is diagnosed with chronic hyperglycemia (heightened levels of blood glucose or blood sugar over the long term), linked to impaired insulin secretion, insulin action or both (David and Gardner, 2011). The progression of its complications is marked by retinopathy, neuropathy, and nephropathy (Wu et al., 2016). Prompt response to this chronic disease is demanded to avert it from deteriorating to severe damage to vital organs and nerve tissues. The α-amylase and α-glucosidase are two important enzymes found in the gastrointestinal tract that involved in the digestion of complex carbohydrates, thus contributing to postprandial hyperglycemia which is a serious complication associated with type 2 diabetes (Shim et al., 2003). Combating hyperglycemia through inhibition of α-amylase and α-glucosidase minimizes digestion and uptake of carbohydrates after food intake, thereby controlling the blood glucose excursion in type 2 diabetes mellitus (Shim et al., 2003).

Chronic hyperglycemia is usually accompanied by oxidative stress which has been implicated to impair endogenous antioxidant defenses, destruction of pancreatic β-cells, excessive lipid peroxidation and damage to cellular organelles (Oboh et al., 2014). For effective management of diabetes mellitus, antioxidant application could be one of the ways to combat diabetes mellitus. This is why natural inhibitors are being sourced from plants as they are cheaper, less toxic, and readily available unlike their acarbose, miglitol, and voglibose (Sabiu and Ashafa, 2016).

*Carica Papaya,* known as pawpaw (English), is the most cultivated species of the *Caricaceae* family, although considered to be an economic tree because of its fruits, which is common in tropical Africa. The seeds are found in the fruits which are very offensive, peppery, and as such unpalatable when eating as food. Their leaves, fruits, seeds, flowers, and roots parts have been documented in different parts of the world as food and medicinal purposes (Anitha et al., 2018). Although a few scientific investigations have been carried out on the *Carica papaya* seeds on its anti-diabetic property. Venkateshwarlu et al. (2015) in their *in vivo* study credited substantial hypoglycemic properties to the seed extract of the plant but the scientific backing to confirm its effectiveness and its possible mode of action is still lacking. Therefore, this study aimed to document the *in vitro* and *in vivo* inhibitory effects of *Carica papaya* seed on α-amylase and α-glucosidase enzymes.

## 2 Materials and methods

### 2.1 Chemicals and reagents

Porcine pancreatic α-amylase, alpha-glucosidase from *Saccharomyces cerevisiae*, 3,5-dinitro salicylic acid (DNSA color reagent), Soluble potato starch, p-nitrophenyl- α -D-glucopyranoside (p-NPG), Streptozotocin 500mg (STZ), sodium phosphate monobasic anhydrous, sodium phosphate dibasic anhydrous, 2,2-diphenyl-1- picrylhydrazyl (DPPH), thiobarbituric reactive substance (TBARS), trichloroacetic acid (TCA), dimethyl sulphoxide (DMSO), ferric chloride, sodium hydroxide pellets, hydrochloric acid, sodium bicarbonate, potassium chloride, sodium acetate trihydrate, obtained from Bristol Scientific Company (Sigma-Aldrich), Lagos, Nigeria. Acarbose (Glucobay) obtained from Gabbyto pharmacy, Yola, Nigeria. All other chemical reagents used in this study were of annular grade.

### 2.2 Plant material

The fruits of *Carica papaya* were collected from farmland at Ofuorachi Igalamela-Odolu, Kogi state, Nigeria. Latitude: 7° 6′ 24″ N. Longitude: 6° 48′ 49″ E. The plant was authenticated by a botanist at the Department of Plant Sciences, School of Life Sciences, Moddibo Adama University of Technology Yola, Nigeria. The ripe seeds were removed and rinsed under running tap water and then shade dried at ambient temperature. Thereafter the dried seed sample was pulverized into a coarse powder using laboratory blender, ready for extraction.

### 2.3 Experimental animals

A total of 240 healthy adult male and female Wistar rats weighing between 90-110 g were obtained from National Veterinary Research Institute Vom, Nigeria after approval of the protocol by the Institutional Animal Ethics Committee. The animals were housed in plastic cages and allowed to acclimatize and feed with vital feeds and water *ad libitum*.

### 2.4 Plant extraction

About (200 g) of powdered *Carica papaya* seed were packed in a soxhlet apparatus and extracted with hexane, ethyl acetate, and methanol sequentially. The extracts obtained were filtered using Whatman filter paper (15 cm) after which the filtrates were concentrated on a rotary evaporator and hexane, ethyl acetate and methanol extracts were obtained. The residue from the methanol extraction using soxhlet extractor was soaked in distilled water for 24 h after which it was filtered using a muslin cloth and Whatman filter paper to obtain the aqueous extract.

### 2.5 Phytochemical screening

Phytochemical screening was performed for all the extracts using standard methods of Trease and Evans (2002); Sofowora (1993); Harbone (1998). The presence of alkaloids, phenols, glycosides, steroids, flavonoids, tannins, saponins, and terpenoids were examined using (Dragendorff and Mayer reagent), (Fehling reagent), (cyanidin reaction), (iron chloride) and (Liebermann-Burchard reaction).

### 2.6 Determination of total phenols by spectrophotometric method

This was done using the method of Edeogal et al. (2005). About 2 g of the extract was boiled with 50 ml of ether for the extraction of the phenolic component for 15 min. Briefly, exactly 5 ml of the extracts was pipette into a 50 ml flask, then about 10 ml of distilled water was added. About 2 ml of ammonium hydroxide solution and 5 ml of concentrated amyl alcohol were also added to the reaction mixture and left to react for 30 min for color formation. This was measured at 505 nm to estimate the total phenolic content using a standard calibration curve that was initially prepared from diluted concentrations of gallic acid. The amount of phenols was calculated as follows:

(1) $$\text{C}=\frac{{\text{C}}_{1}}{\text{N}}\times \text{V}$$

C = Total content of the phenol in mg/g, C_1_ = the concentration of gallic/tannic acid established from the calibration curve of the extract in ml and N = the weight of the plant extract in gm.

### 2.7 Determination of alkaloids

Exactly (5 g) of the sample was weighed into a 250 ml beaker and 200 ml of 10% acetic acid in ethanol was added and covered and allowed to stand for 4 h. This mixture was filtered and the filtrate was concentrated on a water-bath to one-quarter of the original volume. Concentrated ammonium hydroxide was added dropwise to the extract until the preparation was complete. The whole solution was allowed to settle and the precipitate was collected by filtration and weighed (Harbone, 1998). The amount of alkaloids was calculated as follows:

(2) $$\%\;Alkaloid=\frac{final\;weight\;of\;the\;sample}{initial\;weight\;of\;extract}\times 100$$

### 2.8 Determination of saponins

These were done according to the method by Obadoni and Ochuko (2001). The powdered samples about 20 g were put into a conical flask and exactly 100 cm^3^ of 20% aqueous ethanol were added. The samples were heated over a hot water bath for 4 h with continuous stirring at about 55 °C. The solution was filtered and the residue extracted again with another 200 ml 20% ethanol. The extracts were reduced to 40 ml over a water bath at about 90 °C. The 250 ml separatory funnel was used to collect the condensed form of the extract and 20 ml of diethyl ether was added and shaken vigorously. The extract was again washed twice with 10 ml of 5% aqueous sodium chloride. The remaining mixture was heated in a water bath to evaporate the sample and finally dried in the oven to constant weight; the saponin content was calculated as a percentage.

(3) $$\%\;Saponins=\frac{wt\;of\;final\;filtrate}{wt\;of\;sample}\times 100$$

### 2.9 Determination of flavonoids

This determination was done by the method reported by Boham and Kocipai-Abyazan (1994). Exactly 10 g of the seed extract was extracted repeatedly with 100 ml of 80% aqueous methanol at room temperature. The mixture was filtered with Whatman No.42 filter paper, and then the filtrate was collected into a crucible and evaporated into dryness on a water bath and allowed to dry to a constant weight. The amount of flavonoids was calculated as follows:

(4) $$\text{\%}\;Flavonoids=\frac{weight\;of\;final\;filtrate}{wt\;of\;sample}\times 100$$

### 2.10 DPPH (2,2-diphenyl-l-picryl hydrazyl) radical scavenging assay

This was carried out following the method of Lee et al. (2003). Exactly (0.004% w/v) of the DPPH solution was prepared in 95% methanol. A stock solution of extracts and standard ascorbic acid were prepared in the concentration of 10 mg/100 ml (100 μg/ml). From stock solution 2 ml, 4 ml, 6 ml, 8 ml & 10 ml of this solution were taken in five test tubes respectively. With the same solvent made the final volume of each test tube up to 10 ml whose concentration was then 20 μg/ml, 40 μg/ml, 60 μg/ml, 80 μg/ml & 100 μg/ml respectively. About 2 ml of freshly prepared DPPH solution (0.004% w/v) was added in each of these test tubes. The reaction mixture was left standing in the dark for 15 min and thereafter the optical density was recorded at 523 nm against the blank. For the control, 2 ml of DPPH solution in ethanol was mixed with 10 ml of methanol and the optical density of the solution was recorded after 30 min. The assay was carried out in triplicate. The ability of the extract to scavenging DPPH radical was calculated as percentage inhibition (%) using the formula;

(5) $$\text{DPPH}\;\text{Scavenged}\;\left(\text{\%}\right)\;\text{Inhibition}=\frac{\text{Absorbance}\;\text{of}\;\text{control}-\text{Absorbance}\;\text{of}\;\text{test}}{\text{Absorbance}\;\text{of}\;\text{control}}\times 100$$

Where "control" (absorbance of blank) and "test" (absorbance of the test sample).

### 2.11 Thiobarbituric acid (TBA) method

The method of Sawarkar et al. (2011) was used to perform this assay. Briefly, 2 ml of 20% trichloroacetic acid (TCA) and 2 ml of 0.67% TBA solutions were exactly added to 2 ml of the mixtures containing 4 mg of the sample in 4 ml of 99.5% methanol (final concentration 0.02%). This mixture was kept in a water bath (100 °C) for 10 min and after cooling to room temperature, was centrifuged at 3000 rpm for 20 min. The absorbance of the reaction mixture was measured at 532 nm, and antioxidant activity was calculated.

$$\left(\text{\%}\right)\;\text{antioxidant}\;\text{activity}=\frac{\text{Absorbance}\;\text{of}\;\text{control}-\text{Absorbance}\;\text{of}\;\text{test}}{\text{Absorbance}\;\text{of}\;\text{control}}\times 100$$

### 2.12 Ferric reducing antioxidant power (FRAP assay)

In this assay, 1 ml of solvent extracts in different concentration (20 μg/ml, 40 μg/ml, 60 μg/ml, 80 μg/ml & 100 μg/ml respectively) were mixed with 1 ml of 0.2M sodium phosphate buffer (pH 6.6) and 1 ml of 1% potassium ferricyanide in separate test tubes. The reaction mixtures were incubated in a temperature-controlled water bath at 50 °C for 20 min, followed by addition of 1 ml of 10% trichloroacetic acid. The mixtures were then centrifuged for 10 min at room temperature. The supernatant obtained (1 ml) was added with 1 ml of deionized water and 200 μL of 0.1% FeCl3. The blank was also prepared but instead, 1% potassium ferricyanide was replaced by distilled water. The absorbance of the reaction mixture was measured at 700 nm (Banerjee et al., 2008). The percentage reducing power was calculated as,

$$\left(\text{\%}\right)\;\text{antioxidant}\;\text{activity}=\frac{\text{Absorbance}\;\text{of}\;\text{control}-\text{Absorbance}\;\text{of}\;\text{test}}{\text{Absorbance}\;\text{of}\;\text{control}}\times 100$$

### 2.13 Determination of IC_50_ values

IC_50_ values are the concentration of extract at 50% that will inhibit the enzyme's activity and neutralize the reactive oxygen species. It is calculated by plotting percentage inhibition of enzymes and reactive oxygen species against extract concentration in (mg/ml).

### 2.14 *In vitro* determination of alpha-glucosidase inhibitory activity

This assay was carried out following the method of Kim et al. (2005). Briefly, a substrate solution para-nitrophenyl-α-D- glucopyranoside (pNPG) was prepared in 100 mM phosphate buffer (pH 6.9). The substrate (10 ml) was prepared by weighing 60.25 mg of pNPG into a 10 ml volumetric flask and making up the volume to the 10 ml mark with phosphate buffer (pH 6.9). The solution of the extracts was prepared by using an equal volume of distilled water and dimethyl sulphoxide (DMSO). Exactly 10 mg of the extract was weighed into a 10 ml volumetric flask and 5 ml of DMSO was added to dissolve the extract, after which the volume was made up to the 10 ml mark with distilled water to obtain extract stock solution of 1 mg/ml. This stock solution was then diluted to obtain the different concentrations used for the assay. Then 50 μL of α-glucosidase (1U/ml) was added to five tubes containing 20 μL of different concentrations of the extract. After this, 30 μL of 5.0 mM (pNPG) was added to start the reaction. The reaction mixture was incubated at 37 °C for 1 h and stopped by adding 1 ml of 0.1M Na2CO3. The yellow-colored of para-nitrophenol released from pNPG at 400 nm was measured to determine the α-glucosidase activity. The blank was prepared by adding the Na2CO3 to the reaction mixture before adding the enzyme. The results were expressed as a percentage of the negative control in which the extract was replaced with DMSO and distilled water mixture in equal volume. Standard drug (acarbose) was used as the positive control. All the determinations were conducted in triplicates. The enzyme activity expressed as (%) inhibition was calculated as follows:

$$\left(\text{\%}\right)\;\text{Inhibition}=\frac{\text{Absorbance}\;\text{of}\;\text{control}-\text{Absorbance}\;\text{of}\;\text{test}}{\text{Absorbance}\;\text{of}\;\text{control}}\times 100$$

Where “Absorbance of control” (without the extracts) and “Absorbance of test” (with the extracts) are changes in the absorbance reading. The concentrations of *Carica papaya* seed extract causing 50% inhibition of enzyme activity (IC_50_) were determined graphically using the concentration/inhibition curve.

### 2.15 *In vitro* determination of alpha-amylase inhibitory activity

The α-amylase inhibitory activity was assayed following the method of Mccue and Shetty (2004). Briefly, a solution of the enzyme (1 mg/ml) was prepared using 0.02M sodium phosphate buffer (pH 6.9). Starch solution (1%) was also prepared using 0.02M sodium phosphate buffer (pH 6.9) and this was used as a substrate. The solution of the extracts was prepared by using an equal volume of distilled water and dimethyl sulphoxide (DMSO). Exactly 10 mg of the extract was weighed into a 10 ml volumetric flask and 5 ml of DMSO was added to dissolve the extract, after which the volume was made up to the 10 ml mark with distilled water to obtain extract stock solution of 1 mg/ml. This stock solution was then diluted with distilled water to obtain the different concentrations used for the assay.

A total of 50 μL of the extract was added to 50 μL of the alpha-amylase solution, after which 50 μL of 1% starch solution was added and then incubated at 25 °C for 30 min. The reaction was terminated after incubation by adding 100 μL of dinitrosalicylic acid (DNS) reagent. The tubes were then incubated in boiling water for 5 min and cooled to room temperature. The reaction mixture was diluted with 1ml distilled water and the absorbance was measured at 540 nm using a spectrophotometer. The blank and standard were also prepared using the same procedure replacing the extract with distilled water or standard drug. The Standard drug (acarbose) was used as a positive control and the α-amylase inhibitory activity was then calculated.

$$\left(\text{\%}\right)\text{ Inhibition}=\;\frac{\text{Absorbance of control }-\text{ Absorbance of test }}{\text{Absorbance of control}}\;\times 100$$

The IC_50_ of the extract was determined graphically to know its potency.

### 2.16 Induction of hyperglycemia in Wistar rats

The method described by Jin-yin et al. (2012) was used to induce hyperglycemia in the experimental animals after they fasted for 16 h. Streptozotocin (STZ) was dissolved in freshly prepared citrate buffer (0.1 mol/L, pH 4×5) and was intraperitoneally injected to albino rats with a single dose of 50–60 mg/kg/body weight. Fasting blood glucose was monitored for five (5) days using Accu chek glucometer Reader (TERUMO Corporation Ltd., Hatagaya, Tokyo, Japan), and the rats fasted for 16 h after the fifth day. The blood sample was collected from their tails for measurement of blood glucose. Rats with fasting blood glucose higher than 200 mmol/L were considered diabetic and were randomly divided into groups designed.

### 2.17 *In vivo* tolerance tests

#### 2.17.1 Oral glucose tolerance test (OGTT) in diabetic rats

After two weeks adaptation period, the animals were grouped into six (6) groups of five (5) animals each as shown below;

⁃ Group 1: normal control rats (treated with distilled water only)
⁃ Group 2: diabetic control rats (left untreated)
⁃ Group 3: diabetic rats + acarbose (100 mg/kg/body weight)
⁃ Group 4: diabetic rats + extract (250 mg/kg/body weight)
⁃ Group 5: diabetic rats + extract (500 mg/kg/body weight)
⁃ Group 6: diabetic rats + extract (1000 mg/kg/body weight)

The reported method of Subramanian and Asmawi (2008) was adopted. In this test, overnight-fasted rats were treated with different concentrations of the n-hexane, ethyl acetate, methanol and aqueous extracts of *Carica papaya* seed (250, 500 and 1000 mg/kg/body weight), standard drug (acarbose) and distilled water orally. Ten minutes after, the rats were administered glucose (4 g/kg/body weight) orally and blood was collected via tail puncture for blood glucose estimation immediately after the administration of the glucose solution and at 30, 60 and 120 min after glucose treatment. The recorded blood glucose concentrations, peak blood glucose (PBG) and area under the curve (AUC) were determined. Whereas the maximum blood glucose concentration for each group was taken as PBG for the group,

AUC (mg/dL. H) = (BG0 + BG30 x 0.5)/2 + (BG30 + BG60 × 0.5)/2 + (BG60 + BG120 x 1.0)/2

Where the BG_0_ is the blood glucose level before oral administration of glucose and BG_30,_ BG_60,_ and BG_120_ are the blood glucose levels 30, 60 and 120 min after the administration. The same procedure for (OGTT) in diabetic rats was repeated for the (OGTT) non-diabetic rats.

#### 2.17.2 Oral glucose tolerance test (OGTT) in normal rats

The animals were grouped into six (6) groups of five (5) animals each as shown below;

⁃ Group 1: normal control rats (treated with distilled water)
⁃ Group 2: rats + glucose control (left untreated)
⁃ Group 3: rats + acarbose (100 mg/kg/body weight) + glucose
⁃ Group 4: rats + extract (250 mg/kg/body weight) + glucose
⁃ Group 5: rats + extract (500 mg/kg/body weight) + glucose
⁃ Group 6: rats + extract (1000 mg/kg/body weight) + glucose

The reported method of Subramanian and Asmawi (2008) was adopted. In this test, overnight-fasted rats were treated with different concentrations of the n-hexane, ethyl acetate, methanol and aqueous extracts of *Carica papaya* seed (250, 500 and 1000 mg/kg/body weight), acarbose and distilled water orally. Ten minutes after, the rats were administered glucose (4 mg/kg/body weight) orally and blood was collected via tail puncture for blood glucose estimation immediately after the administration of the glucose solution and at 30, 60 and 120 min after glucose treatment. The recorded blood glucose concentrations, peak blood glucose (PBG) and area under the curve (AUC) were determined. Whereas the maximum blood glucose concentration for each group was taken as PBG for the group, where the BG_0_ is the blood glucose level before oral administration of glucose and BG_30,_ BG_60,_ and BG_120_ are the blood glucose levels 30, 60 and 120 min after the administration. The same procedure for (OGTT) in diabetic rats was repeated for the (OGTT) non-diabetic rats.

### 2.18 GC-MS analysis

Hexane and ethyl acetate extracts of *Carica papaya* seed were analyzed with the help of a GC-MS analyzer (GC-MS-QP 2010 plus Shimadzu, Japan). The carrier gas helium (99.999 %) was used at a flow rate of 1 ml per min in split mode (10:1) v/v. Methanol and aqueous extracts (8 μL) were injected into the column at 250 °C injector temperature. The temperature of the oven started at 70 °C and held for 5 min. It was then raised at a rate of 10 °C per min to 280 °C without holding. Holding was allowed for 6 min at a programmed rate of 5 °C per min. The temperature of ion sources was maintained at 200 °C. The injector temperature was set at 250 °C and the detector temperature was set at 250 °C. The mass spectrum of compounds present in samples was obtained by electron ionization at 70 eV and the detector operates in scan mode 50–600 Da atomic units. The MS Table was generated through an ACQ mode scan within 0.5 s of scan interval at the speed of 666 and fragments from 30 to 350 Da were maintained. The total running was 21 min.

The compounds identification was done by direct spectrum comparison of the retention times, mass spectral data, and fragmentation pattern of the unknown constituents of the extract was made with those in the National Institute of Standard and Technology (NIST) libraries having more than 62,000 compounds.

### 2.19 Fourier transformed infrared (FTIR) spectroscopic analysis

Fourier transform infrared spectroscopic (FTIR) analysis of the extracts was carried out using Shimadzu FTIR– 8400s Fourier transform infrared spectrophotometer, Japan. Hexane and ethyl acetate extracts of *Carica papaya* seed were oven-dried to get powders of the different solvent extracts used for FTIR analysis. The dried extracts powder (10 mg) were encapsulated in 100 mg of KBr pellet, to prepare translucent sample disc and analysis was carried out by scanning the samples through a wave number range of 400–4000 cm-1 with a resolution of 2 cm-1. The peak values of FTIR were recorded and possible chemical interactions were examined.

### 2.20 Statistical analysis

The results are presented as Mean ± SEM (Standard Error of Mean). Comparisons between the groups were performed by one-way analysis of variance using Statistical Package for Social Sciences (SPSS) for windows version 28.0 (SPSS Inc., Chicago, IL, USA). Significant differences were compared by Duncan's Multiple Range test; a probability level of less than 5% (P < 0.05) was considered significant.

## 3 Results

### 3.1 Phytochemical screening of seeds extracts

Table 1 shows the preliminary phytochemical components detected in the solvent extracts of *Carica papaya* seed. The methanol and aqueous seed extracts contained more phytochemical components than hexane and ethyl acetate extracts. The percentage composition of some phytochemical components detected in the extracts of *Carica papaya* seed was analyzed in Table 2. These revealed a higher amount of saponins, flavonoids, and alkaloids in the extracts as depicted in Table 2.

**Table 1:** Phytochemical components detected in the crude extracts of *Carica papaya* seed.

Extract	Saponins	Tannins	Terpenoids	Flavonoids	Alkaloids	Glycosides	Steroids	Phenols
Hexane	+	-	-	-	+	-	-	+
Methanol	+	-	+	+	+	-	+	+
Ethyl acetate	+	-	-	+	-	-	-	+
Aqueous	+	-	+	+	+	-	-	+

Key: +: Present; -: Absent.

**Table 2:** Percentage composition of some phytochemical components in the crude extracts (%).

Extract	Saponins	Tannins	Terpenoids	Flavonoids	Alkaloids	Glycosides	Steroids	Phenols
Hexane	2.41 ± 0.09	ND	ND	ND	1.54 ± 0.03	ND	ND	0.08 ± 0.03
Methanol	1.93 ± 0.01	ND	0.11 ± 0.01	1.78 ± 0.08	1.27 ± 0.02	ND	0.05 ± 0.02	0.21 ± 0.06
Ethylacetate	2.16 ± 0.50	ND	ND	0.29 ± 0.07	ND	ND	ND	0.50 ± 0.02
Aqueous	1.57 ± 0.07	ND	0.06 ± 0.01	1.33 ± 0.12	2.01 ± 0.06	ND	ND	0.34 ± 0.04

Values are Mean ± SD, ND: Not Detected.

### 3.2 *In vitro* antioxidant capacity of the *Carica papaya* seed extracts

The DPPH (2, 2-diphenyl-l-picryl hydrazyl) free radical scavenging ability of *Carica papaya* seed extracts was determined and the result is presented in Table 3. The result revealed that all the extracts scavenged DPPH (2, 2-diphenyl-l-picryl hydrazyl) free radical in a dose-dependent manner (20–100 mg/ml). However, the hexane extract (71.39 ± 0.59^b^ to 92.33 ± 0.52^ab^) had the highest DPPH free radical scavenging ability, whereas the aqueous extract (50.23 ± 0.33^b^ to 86.28 ± 0.33^b^) had the least comparable to standard (8.18 ± 0.34 to 58.62 ± 0.69) (Table 3).

**Table 3:** DPPH radical scavenging activity (% Inhibition) of *C. papaya* seed extracts.

Concentration	Hexane	Methanol	Ethylacetate	Aqueous	L-Ascorbic (standard)
20	71.39 ± 0.59^b^	76.33 ± 0.41^a^^b^	74.19 ± 0.93^b^	50.23 ± 0.33^b^	8.18 ± 0.34
40	83.02 ± 0.84^a^^b^	80.27 ± 0.38^b^	79.73 ± 0.25^b^	76.52 ± 0.14^b^	17.05 ± 0.01
60	88.89 ± 1.34^a^^b^	85.38 ± 0.37^b^	85.68 ± 0.31^b^	79.89 ± 0.16^b^	48.82 ± 0.02
80	90.05 ± 0.54^a^^b^	88.73 ± 0.38^b^	87.85 ± 0.50^b^	82.39 ± 0.05^b^	49.53 ± 0.06
100	92.33 ± 0.52^a^^b^	89.95 ± 0.09^b^	89.73 ± 0.10^b^	86.28 ± 0.33^b^	58.62 ± 0.69
IC_50_ (mg/ml)	41.48	42.38	42.61	45.68	80.89

Values are Mean ± SD (n = 3).

a Significantly (p < 0.05) higher compared to other extract at the same concentration.

b Significantly (p < 0.05) higher compared to L-ascorbic acid at the same concentration.

The TBA (thiobarbituric acid) scavenging ability of *Carica papaya* seed extracts was determined and the result is presented in Table 4. The IC_50_ (extract concentration that will inhibit and neutralize 50% oxidative potential of reactive oxygen species) revealed that the hexane extract (IC_50_ = 38.22 mg/ml) had the highest TBA scavenging ability followed by methanol extract (IC_50_ = 40.84 mg/ml) while the aqueous extract (IC_50_ = 44.00 mg/ml) had the least activity when compared to other extracts and ascorbic acid (standard drug) as shown in Table 4.

**Table 4:** TBA radical scavenging activity (% Inhibition) of *C. papaya* seed extracts.

Concentration	Hexane	Methanol	Ethylacetate	Aqueous	L-Ascorbic (standard)
20	95.56 ± 0.03^a^^b^	81.48 ± 1.81^b^	87.73 ± 0.11^b^	70.64 ± 0.07^b^	40.65 ± 0.75
40	95.97 ± 0.02^a^^b^	82.91 ± 0.21^b^	86.11 ± 0.28^b^	81.65 ± 0.24^b^	42.70 ± 0.23
60	95.82 ± 0.08^a^^b^	87.17 ± 0.47^b^	87.42 ± 0.11^b^	82.45 ± 0.44^b^	55.54 ± 1.40
80	95.98 ± 0.01^a^^b^	92.57 ± 0.04^b^	88.11 ± 0.01^b^	85.18 ± 0.16^b^	57.02 ± 2.45
100	96.03 ± 0.01^a^^b^	93.51 ± 0.01^b^	85.50 ± 0.30^b^	85.61 ± 0.14^b^	52.71 ± 0.79
IC_50_ (mg/ml)	38.22	40.84	42.24	44.00	70.13

Values are Mean ± SD (n = 3).

a Significantly (p < 0.05) higher compared to other extract at the same concentration.

b Significantly (p < 0.05) higher compared to L-ascorbic acid at the same concentration.

FRAP (Ferric reducing antioxidant power) of *Carica papaya* seed extracts was also determined and the result obtained is presented in Table 5. As concentration increases (20–100 mg/ml), ferric reducing antioxidant power of *Carica papaya* seed extract also increases but ethyl acetate extract (96.00 ± 0.15) at 100 mg/ml had the highest inhibition. The IC_50_ values revealed that ethyl acetate extract (IC_50_ = 38.75 mg/ml) is the most potent extract compared to other extracts and ascorbic acid as shown in Table 5.

**Table 5:** Ferric reducing antioxidant power (FRAP) of *C. papaya* seed extracts (% Inhibition).

Concentration	Hexane	Methanol	Ethylacetate	Aqueous	L-Ascorbic (standard)
20	84.85 ± 0.18	71.15 ± 0.06	89.79 ± 0.12^a^	84.79 ± 0.01	90.33 ± 0.04
40	90.48 ± 0.53	89.44 ± 0.42	91.76 ± 0.06^a^	87.30 ± 0.07	92.21 ± 0.29
60	94.23 ± 0.30	93.35 ± 0.07	94.52 ± 0.02^b^	90.92 ± 0.03	93.09 ± 0.01
80	94.34 ± 0.17	94.25 ± 0.06	95.53 ± 0.04^a^^,^^b^	94.20 ± 0.01	94.52 ± 0.01
100	95.23 ± 0.08	95.19 ± 0.07	96.00 ± 0.15^a^^,^^b^	94.93 ± 0.04	95.24 ± 0.02
IC_50_ (mg/ml)	39.23	39.77	38.75	39.75	39.05

Values are Mean ± SD (n = 3).

a Significantly (p < 0.05) higher compared to other extract at the same concentration.

b Significantly (p < 0.05) higher compared to L-ascorbic acid at the same concentration.

### 3.3 *In vitro* inhibitory effects of the extracts of *Carica papaya* seed on α-amylase and α-glucosidase

The inhibitory effect of *Carica papaya* seed extracts on α-amylase activity is presented in Table 6. The result revealed that all the extracts inhibited α-amylase in a dose-dependent pattern (20–100 mg/ml), however, as revealed by the IC_50_ values Table 6, hexane extract of *Carica papaya* seed (IC_50_ = 76.96 mg/ml) had the highest inhibitory effect on α-amylase activity followed by ethyl acetate (IC_50_ = 79.18 mg/ml) while the methanol extract (IC_50_ = 94.63 mg/ml) had the least activity comparable to standard (74.64 mg/ml). On the other hand, the ability of the extracts to inhibit α-glucosidase activity *in vitro* was also investigated and the result is presented in Table 7. All the extracts exhibited a dose-dependent enzyme inhibitory activity, however, the hexane extract (IC_50_ = 75.78 mg/ml) elicited the most potent and prominent effect compared to other extracts Table 7.

**Table 6:** Inhibition of α-amylase activity by *Carica papaya* seed extracts (% Inhibition).

Concentration	n-hexane	Ethylacetate	Methanol	Aqueous	Acarbose (standard)
20	23.04 ± 1.82^a^	22.52 ± 6.06	21.86 ± 0.72	18.52 ± 0.76	23.63 ± 0.33
40	37.37 ± 2.33^a^^b^	33.61 ± 1.56^b^	26.47 ± 4.73	21.43 ± 2.58	31.39 ± 1.65
60	48.66 ± 1.79^a^^b^	38.26 ± 3.18	26.41 ± 2.45	23.20 ± 1.01	42.49 ± 4.63
80	51.15 ± 3.71^a^	49.85 ± 5.92	41.53 ± 6.40	44.37 ± 1.77	57.38 ± 1.62
100	53.27 ± 0.10	58.12 ± 0.18^a^	52.22 ± 7.90	56.25 ± 1.87	58.69 ± 4.10
IC_50_ (mg/ml)	76.96	79.18	94.63	93.26	74.64

Values are Mean ± SD (n = 3).

a Significantly (p < 0.05) higher compared to other extracts at the same concentration.

b Significantly (p < 0.05) higher compared to Acarbose at the same concentration.

**Table 7:** Inhibition of α-glucosidase activity by *Carica papaya* seed extracts (% Inhibition).

Concentration	n-hexane	Ethyl acetate	Methanol	Aqueous	Acarbose (standard)
20	20.09 ± 0.79	20.79 ± 6.97	24.31 ± 2.25^a^	19.03 ± 2.74	25.87 ± 2.04
40	23.64 ± 1.12	30.20 ± 1.92	35.57 ± 1.72^a^	26.12 ± 1.91	37.64 ± 0.88
60	33.27 ± 0.79	31.56 ± 4.25	41.80 ± 2.37^a^	34.48 ± 0.63	46.71 ± 13.56
80	58.60 ± 0.95^a^^b^	55.80 ± 1.96	43.48 ± 1.25	38.76 ± 1.19	54.09 ± 1.61
100	64.84 ± 1.10^a^^b^	62.28 ± 1.19	59.67 ± 1.89	41.48 ± 14.85	62.38 ± 3.47
IC_50_ (mg/ml)	75.78	77.41	79.35	102.40	71.47

Values are Mean ± SD (n = 3).

a Significantly (p < 0.05) higher compared to other extracts at the same concentration.

b Significantly (p < 0.05) higher compared to Acarbose at the same concentration.

### 3.4 *In vivo* inhibitory effects of the crude extracts of *Carica papaya* seed

**Figure 1:**
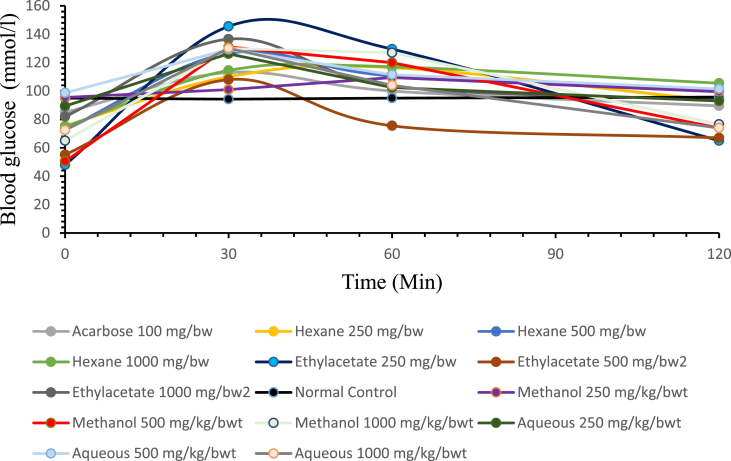
Effect of extract of *Carica papaya* seed extracts on oral glucose tolerance test in normal rats.

**Figure 2:**
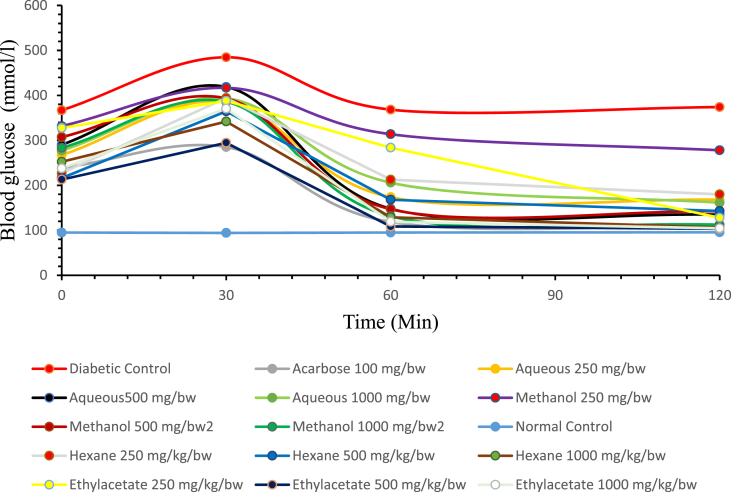
Effect of *Carica papaya* seed extracts on oral glucose tolerance test in diabetic rats.

Oral glucose tolerance test (OGTT) of the *Carica papaya* seed extracts in normal and streptozotocin-induced diabetic rats is presented in (Figures 1 and 2). Figure 1 shows the changes in the blood glucose level of the normal rats (mmol/L) that were administered with glucose in the presence of the crude extracts of *Carica papaya* seed for 120 min. The initial blood glucose at (0 min) is the blood glucose level of normal rats that have not been administered with glucose load. The highest increase in blood glucose level was observed at (30 min) after the glucose load in all the treatment groups. Thereafter, the blood glucose level decreased over a 120 min time interval to a level close to the baseline at (0 min). From the curve, it was observed that ethyl acetate extract (250 and 500 mg/kg/body weight) and aqueous extract (1000 mg/kg/body weight) had the most significant (P < 0.05) reduction when compared with the other extracts at the same concentration and the acarbose (standard drug) (Figure 1).

Figure 2 shows the changes in the blood glucose level of streptozotocin-induced diabetic rats (mmol/L). The initial baseline blood glucose level at (0 min) is the blood glucose level of rats that have been induced with hyperglycemia and confirmed diabetic before administration of the extracts and glucose load. The diabetic control group (DC) was left untreated and the acarbose control group served as the standard drug while other groups were treated with the extracts (250, 500 and 1000 mg/kg/body weight). The blood glucose level at 30 min was elevated after the glucose load and decreased over time, in all the groups of diabetic rats. From the curve, it was observed that the ethyl acetate extract (500 and 1000 mg/kg/body weight) reduced the blood glucose level of the diabetic rats over a period of 120 min in a dose-dependent manner significantly (P < 0.05) lower even better than the reference drug (acarbose) while other extracts decreased blood glucose level close to normal when compared (Figure 2).

### 3.5 GC-MS chromatogram of hexane and ethyl acetate extracts

**Figure 3:**
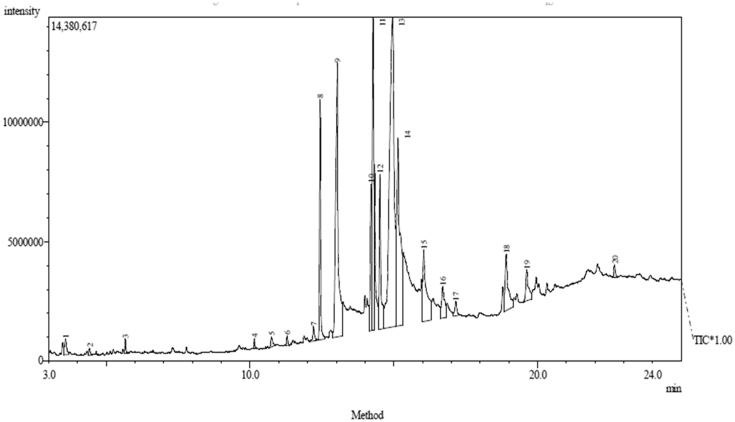
GC-MS Chromatogram of hexane extract of the *Carica papaya* seed.

**Figure 4:**
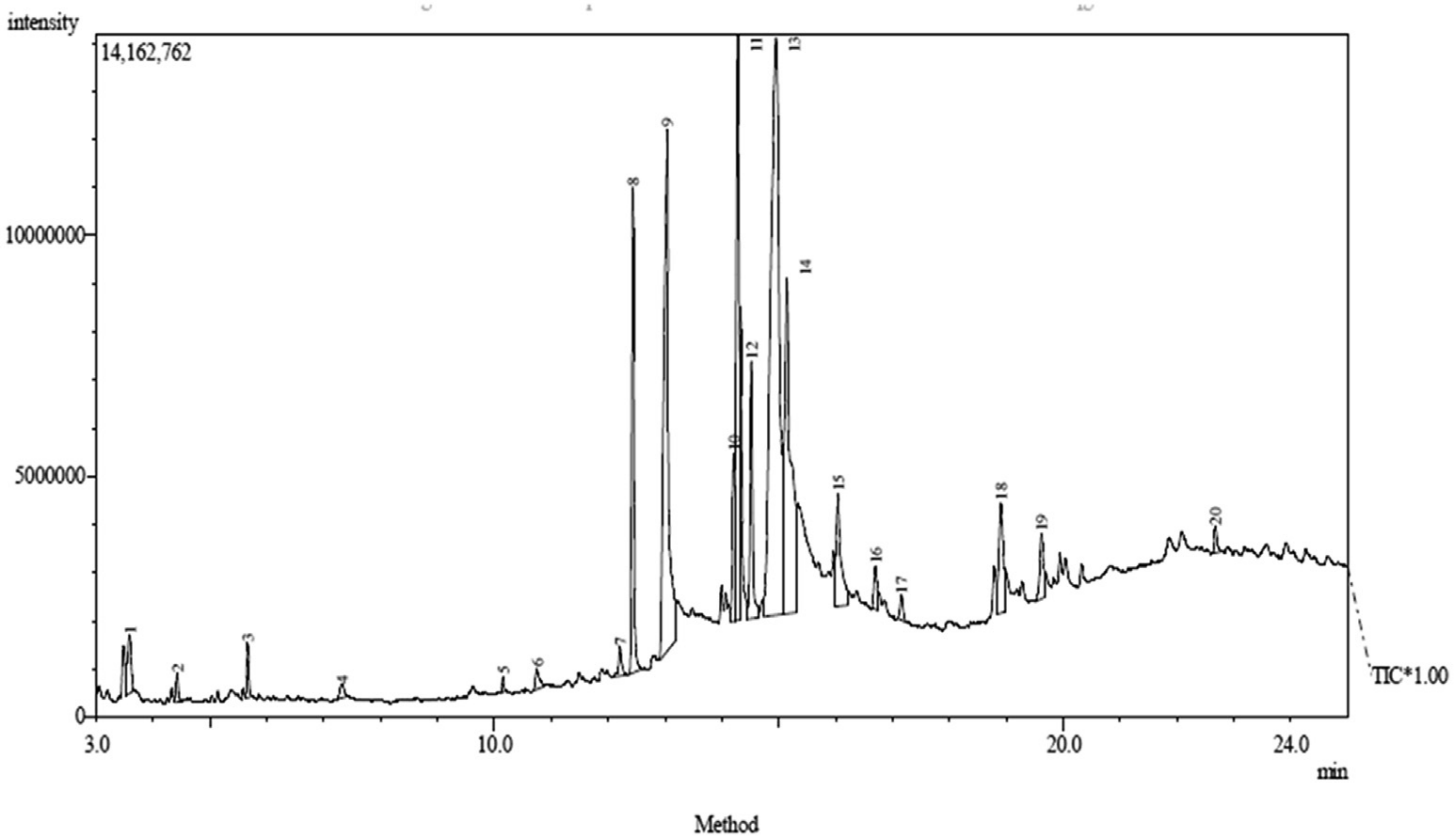
GC-MS chromatogram of ethyl acetate extract of the *Carica papaya* seed.

Since the hexane extract elicited the most potent inhibitory effect followed by ethyl acetate extract, they were subjected to GC-MS profiling as depicted in Figures 3 and 4. The identified compounds in the chromatograms of hexane and ethyl acetate extracts are presented in Tables 8 and 9. The major compounds present in hexane extract of *Carica papaya* seed are; oleic acid (30.88%), n-hexadecanoic acid (15.51%), octadecanoic acid (12.89%), 11-octadecenoic acid, methyl ester (8**.**95%), pentadecanoic acid, 14-methyl-, methyl ester (5.97%), 1,1,1-Trifluoroheptadecen-2-one (5.70%) and octadecanoic acid methyl ester (5.22%) while oleic acid (32.17%), n-hexadecanoic acid (16.44%), Octadecanoic acid (13.40%), 11-octadecenoic acid, methyl ester (10**.**56%), Pentadecanoic acid, 14-methyl-, methyl ester (7.11%) and octadecanoic acid methyl ester (%) were found in ethyl acetate extract.

**Table 8:** GC-MS Identified components of the hexane extract of the seed of *Carica papaya*.

S/N	Retention time	Peak area (%)	Formula	Molecular weight	Compound name
1	3.582	0.71	C_8_H_14_O	126	2-hexenal, 2-ethyl-
2	4.421	0.18	C_6_H_14_O_2_	118	Propane, 1, 1-dimethoxy-2-methyl-
3	5.668	0.38	C_6_H_14_O_2_	118	Propane, 1, 1-dimethoxy-2-methyl-
4	10.148	0.25	C_15_H_30_O_2_	242	Methyl tetradecanoate
5	10.742	0.44	C_14_H_28_O_2_	228	Tetradecanoic acid
6	11.294	0.25	C_17_H_34_O_2_	270	Isopropyl Myristate
7	12.209	0.50	C_13_H_26_O	198	2-Tridecanone
8	12.434	5.97	C_17_H_34_O_2_	270	Pentadecanoic acid, 14-methyl-, methyl ester
9	13.035	15.51	C_16_H_32_O_2_	256	n-Hexadecanoic acid
10	14.209	3.88	C_19_H_34_O_2_	294	9,12-Octadecadienoic acid, methyl ester, (E,E)-
11	14.279	8.95	C_19_H_36_O_2_	296	11-Octadecenoic acid, methyl ester
12	14.519	5.22	C_19_H_38_O_2_	298	Octadecanoic acid methyl ester
13	14.956	30.88	C_18_H_34_O_2_	282	Oleic acid
14	15.145	12.89	C_18_H_36_O_2_	284	Octadecanoic acid
15	16.042	5.70	C_17_H_31_F_3_O	308	1,1,1-Trifluoroheptadecen-2-one
16	16.702	1.99	C_37_H_74_NO_8_P	691	Palmitin, 1,2-di-, 2-aminoethyl hydrogen phosphate
17	17.157	0.62	C_21_H_42_O_2_	326	Eicosanoic acid, methyl ester
18	18.904	3.56	C_22_H_42_O_2_	338	(E)-13-Docosenoic acid
19	19.620	1.72	C_18_H_34_O	266	9-Octadecenal
20	22.680	0.39	C_25_H_50_O_2_	382	Tetracosanoic acid, methyl ester

**Table 9:** GC-MS Identified components of the ethyl acetate extract of the *Carica papay*a seed.

S/N	Retention time	Peak area (%)	Formula	Molecular weight	Compound name
1	3.585	1.41	C_8_H_14_O	126	2-hexenal, 2-ethyl-
2	4.425	0.39	C_6_H_14_O_2_	118	Propane, 1, 1-dimethoxy-2-methyl-
3	5.668	0.75	C_6_H_14_O_2_	118	Propane, 1, 1-dimethoxy-2-methyl-
4	7.311	0.36	C_10_H_22_O_4_	206	Ethanol, 2-[2-(2-butoxyethoxy)ethoxy]-
5	10.148	0.18	C_15_H_30_O_2_	242	Methyl tetradecanoate
6	10.743	0.53	C_14_H_28_O_2_	228	Tetradecanoic acid
7	12.208	0.55	C_18_H_34_O_2_	282	Cyclopropaneoctanoic acid, 2-hexyl-, methyl ester
8	12.434	7.11	C_17_H_34_O_2_	270	Pentadecanoic acid, 14-methyl-, methyl ester
9	13.034	16.44	C_16_H_32_O_2_	256	n-Hexadecanoic acid
10	14.206	2.24	C_19_H_34_O_2_	294	9,12-Octadecadienoic acid, methyl ester, (E,E)-
11	14.279	10.56	C_19_H_36_O_2_	296	11-Octadecenoic acid, methyl ester
12	14.518	4.03	C_19_H_38_O_2_	298	Octadecanoic acid methyl ester
13	14.951	32.17	C_18_H_34_O_2_	282	Oleic acid
14	15.142	13.40	C_18_H_36_O_2_	284	Octadecanoic acid
15	16.040	3.51	C_16_H_34_N_2_O	270	16-Hexadecanoyl hydrazide
16	16.698	0.91	C_37_H_74_NO_8_P	691	Palmitin, 1,2-di-, 2-aminoethyl hydrogen phosphate
17	17.156	0.44	C_21_H_42_O_2_	326	Eicosanoic acid, methyl ester
18	18.901	2.95	C_19_H_36_O_2_	296	5-Octadecenoic acid, methyl ester
19	19.616	1.58	C_19_H_36_O	280	2-Methyl-Z,Z-3,13-octadecadienol
20	22.680	0.46	C_25_H_50_O_2_	382	Tetracosanoic acid, methyl ester

### 3.6 FTIR spectra data interpretation of the extracts of *Carica papaya* seed

**Figure 5:**
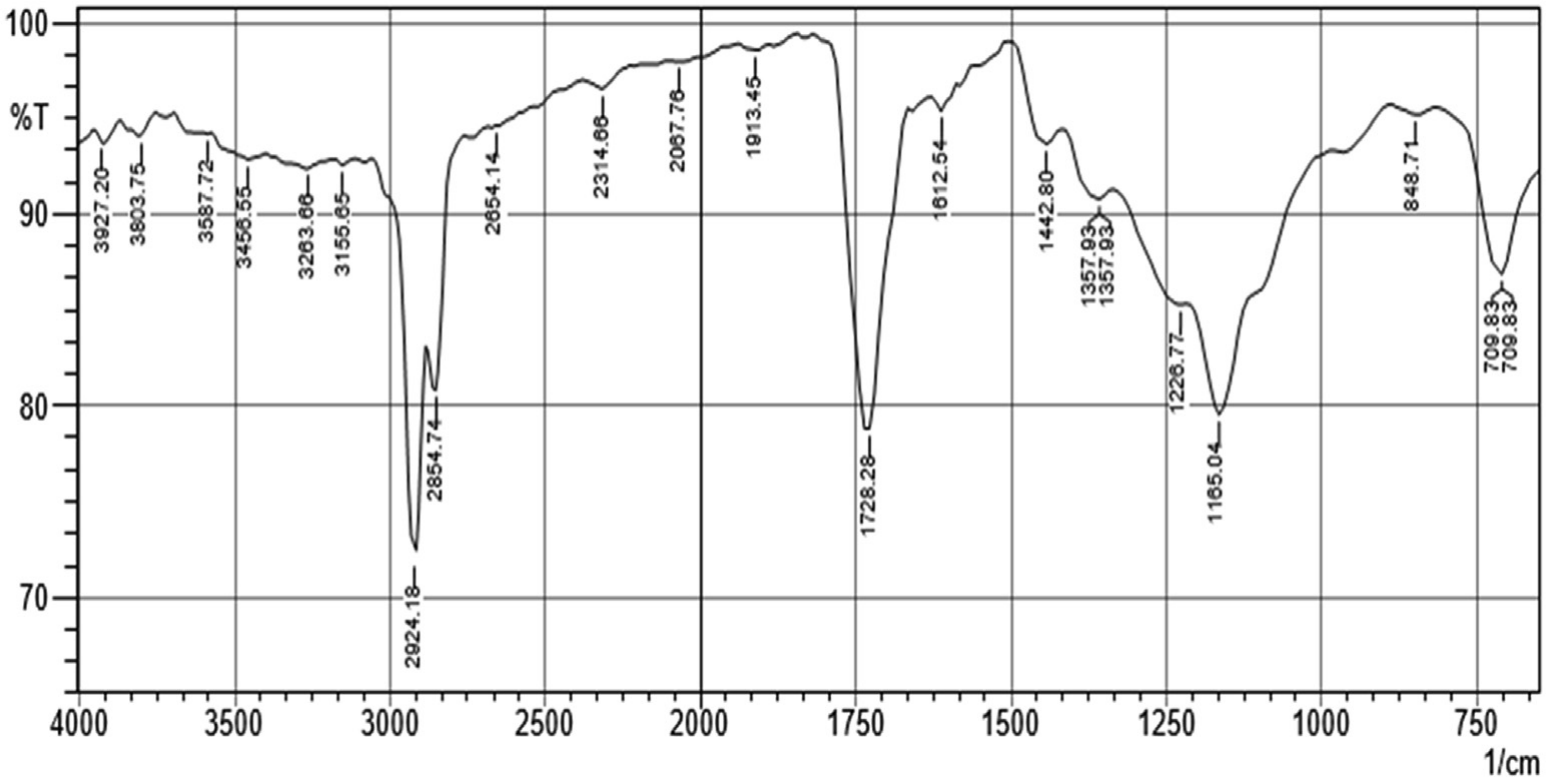
FTIR spectrum of hexane extract of *C. papaya* seed.

**Figure 6:**
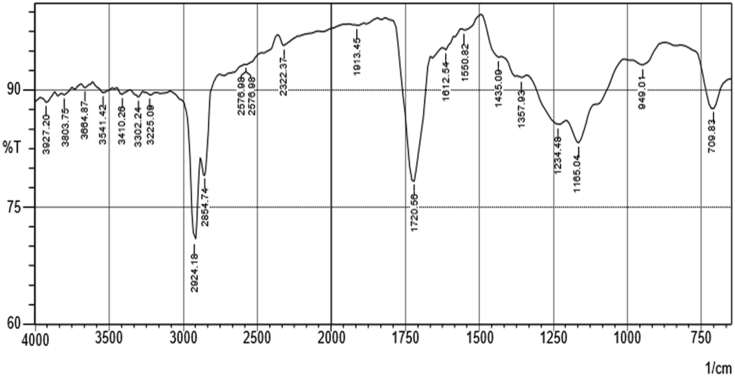
FTIR spectrum of ethyl acetate extract of *C. papaya* seed.

Figure 5 shows the FTIR spectrum of hexane extract of *Carica papaya* seed and the result of FTIR interpretation is presented in Table 10 while the FTIR spectrum of the ethyl acetate extract is presented in Figure 6 and the result of FTIR interpretation is displayed in Table 11. The main functional groups present in hexane and ethyl acetate extracts of *Carica papaya* seed are -C=C, -C-Cl, -C-O, -O-H, -CH, -C=O, -C=C=C, -N=C=S, -O=C=O and -N-H.

**Table 10:** FTIR Interpretation of the n-hexane extract of the *Carica papaya* seed.

S/N	Test sample (cm^−1^)	Reference standard (cm^−1^)	Functional group Assignment	Identified compound
1	709.83	665–730	C=C bend	Alkene
2	709.83	665–730	C=C bend	Alkene
3	848.71	550–850	C-Cl stretch	halo compound
4	1165.04	1163–1210	C-O stretch	ester compound
5	1226.77	1220–1275	C-O stretch	alkyl aryl ether
6	1357.93	1310–1390	O-H bend	Phenol
7	1357.93	1310–1390	O-H bend	Phenol
8	1442.8	1350–1480	C-H bend	Alkane
9	1612.54	1566–1650	C=C stretch	cyclic alkene
10	1728.28	1720–1740	C=O stretch	aldehyde
11	1913.45	1900–2000	C=C=C stretch	allene group
12	2067.76	1990–2140	N=C=S stretch	Isothiocyanate
13	2314.66	2300–2400	O=C=O stretch	carbonate
14	2654.14	2600–2800	C-H stretch	Alkane
15	2854.74	2850–2960	C-H stretch	Alkane
16	2924.18	2850–2960	C-H stretch	Alkane
17	3155.65	3100–3200	O-H stretch	Alcohol
18	3263.66	3200–3400	O-H stretch	Alcohol
19	3456.55	3350–3500	N-H stretch	Amine
20	3587.72	>3500	O-H stretch	Alcohol
21	3803.75	>3500	O-H stretch	Alcohol
22	3927.2	>3500	O-H stretch	Alcohol

**Table 11:** FTIR Interpretation of the ethyl acetate extract of the *Carica papaya* seed.

S/N	Test sample (cm^−1^)	Reference standard (cm^−1^)	Functional group Assignment	Identified Compound
1	709.83	665–730	C=C bend	Alkene
2	949.01	850–995	P-O-C stretch	aromatic phosphates
3	1165.04	1163–1210	C-O stretch	Ester
4	1234.48	1220–1275	C-O stretch	alkyl aryl ether
5	1357.93	1310–1390	O-H bend	phenol
6	1435.09	1395–1440	O-H bend	carboxylic acid
7	1550.82	1500–1550	N-O stretch	nitro compound
8	1612.54	1566–1650	C=C stretch	cyclic alkene
9	1720.56	1720–1740	C=O stretch	Aldehyde
10	1913.45	1900–2000	C=C=C stretch	allene
11	2322.37	2300–2400	O=C=O stretch	Carbonate
12	2576.98	2550–2600	S-H stretch	thiols
13	2576.98	2550–2600	S-H stretch	thiols
14	2854.74	2840–3000	C-H stretch	Alkane
15	2924.18	2840–3000	C-H stretch	Alkane
16	3225.09	3200–3550	O-H stretch	Alcohol
17	3302.24	3200–3550	O-H stretch	Alcohol
18	3410.26	3200–3550	O-H stretch	Alcohol
19	3541.42	3200–3550	O-H stretch	Alcohol
20	3664.87	>3500	O-H stretch	Alcohol
21	3803.75	>3500	O-H stretch	Alcohol
22	3927.2	>3500	O-H stretch	Alcohol

## 4 Discussion

There are limitations associated with potent α-amylase and α-glucosidase inhibitors like acarbose, miglitol, and voglibose, which has mandated researchers to scientifically validate the use of plant extracts to prevent/manage postprandial hyperglycemia that manifested from α-amylase and α-glucosidase activity. The lowering of postprandial hyperglycemia through the use of α-amylase and α-glucosidase inhibitors from plant extracts becomes a critical therapeutic target to ameliorate the progression of diabetes as well as its complications, such as neuropathy, nephropathy, and retinopathy (Joshi et al., 2015). This option potentiate optimization of glucose metabolism over the whole day, as it contributes to the adaptation of insulin secretion.

These plant extracts have therapeutic application via saponins, flavonoids, alkaloids, terpenoids, flavonoids, steroids and phenols (Nair et al., 2013).

The presence of the phytochemical constituents such as saponins, flavonoids, and alkaloids in higher amounts in hexane and ethyl acetate extracts was the highlight of the study as presented in **(**Tables 1 and 2**)**. Previous studies have established the hypoglycemic and antioxidant potentials of these phytochemical constituents (Chiang et al., 2014; Meng et al., 2016). The present study has explicated a remarkable antioxidant potentials of *Carica papaya* seed extracts. *In vitro*, antioxidant assays were carried out to determine a possible known mechanism by which extracts of *Carica papaya* seeds operate to inhibit oxidative stress linked to type 2 diabetes. The hexane extract indicated the most potent DPPH (IC_50_ = 41.5 mg/ml) and TBA (IC_50_ = 38.2 mg/ml) radical scavenging activity respectively when compared with control whereas ethyl acetate extract aligned the most potent ferric reducing antioxidant power with IC_50_ = 38.8 mg/ml. Hexane and ethyl acetate extracts were found to be high in saponins. Thus, the antioxidant activity shown by hexane and ethyl acetate extract can be attributed to its rich saponins contents.

One of the major characteristics of acarbose, miglitol, and voglibose used in the management of Type 2 diabetes is the ability to inhibit α-amylase and α-glucosidase enzymes (Chipiti et al., 2015). The result of the *in vitro* investigation of extracts of *Carica papaya* seed on α-amylase and α-glucosidase revealed that the extracts had a less potent and moderate inhibitory activity when compared with the control (standard). The decrease in the enzyme inhibitory activity exhibited by the extracts was deduced from the IC_50_ (extract concentration causing 50% enzyme inhibition). Thus, hexane extract produced a significant potent inhibitory activity with IC_50_ of 76.9 and 75.8 mg/ml respectively, for α-amylase and α-glucosidase when compared with the other extracts. This is not surprising going by the various therapeutic attributes of *Carica papaya* seed (Basalingappa et al., 2018). These findings suggest that bioactive compounds may be present in *Carica papaya* seed extracts which exerts antidiabetic effect by delaying carbohydrate digestion into absorbable units.

The *in vivo* study has demonstrated that *Carica papaya* seed extracts can reduce postprandial hyperglycemia during the oral glucose tolerance test (OGTT) in normal and streptozotocin-induced diabetic rats. However, this reduction is more evident in ethyl acetate extract than acarbose. All the treatment groups gave a different type of glucose curve in normal and diabetic rats. In the normal rat groups, extracts significantly decrease blood glucose levels at the end of 120 min treatment as presented in Figure 1. However, it was observed from the curve that ethyl acetate extract 250 and 500 mg/kg body weight and aqueous extract 1000 mg/kg body weight have the most significant (P < 0.05) reduction when compared with the other extracts at the same concentration with the acarbose (standard drug). In the diabetic rats, the blood glucose level increased maximally at 30 min treatment with glucose load and decreased at the end of 120 min treatment as presented in Figure 2. However, there was a significant decrease in the group treated with ethyl acetate extract 500 and 1000 mg/kg body weight at the end of treatment as compared to the standard drug and diabetic control group. This has suggested that the dose 500 and 1000 mg/kg body weight of ethyl acetate extract is more effective in the treatment of streptozotocin-induced diabetes mellitus in the rat model than all other doses of extracts. This decrease in blood glucose level could be attributed to some level of *in vivo* inhibition against α-amylase and α-glucosidase resulting in a suppression of hyperglycemia (Ahmed et al., 2015).

Characterization of hexane and ethyl acetate extracts using gas chromatography (GC-MS) as shown in Tables 8 and 9 revealed that oleic acid, n-hexadecanoic acid, octadecanoic acid, 11-octadecenoic acid, methyl ester, and pentadecanoic acid, 14-methyl-, methyl ester as the major bioactive compounds. Previous studies have established the antioxidant and remarkable antidiabetic potentials of these compounds to regenerate pancreatic β-cell and inhibit the activity of the α-glucosidase enzyme (Omotoso et al., 2014; Gnanavel and Saral, 2013; Gomathi et al., 2015; Sheeba and Viswanathan, 2016). The number of peak values depicted by FTIR spectroscopic study of hexane and ethyl acetate extracts has confirmed the presence of functional groups that are indicative of flavonoids and saponins among other compounds. The main functional groups present in hexane and ethyl acetate extracts of *Carica papaya* seed are: -C=C, -C-Cl, -C-O, -O-H, -CH, -C=O, -C=C=C, -N=C=S, -O=C=O and -N-H (Tables 5 and 6). The –OH group in the chemical structure of saponins and flavonoids has been linked to antioxidant and inhibitory processes (Maobe and Nyarango, 2013). This result supports earlier reports of the antidiabetic potential of different parts of unripe pawpaw fruit *in vitro* (Oboh et al., 2014).

## 5 Conclusion

It was concluded that *Carica papaya* seeds have shown remarkable inhibitory activity against α-amylase and α-glucosidase enzymes and oxidative stress linked with diabetes, which could be the possible mechanisms by which they lower blood sugar levels to manage type 2 diabetes mellitus. However, these characteristics may be due to the bioactive compounds such as oleic acid, n-hexadecanoic acid, octadecanoic acid, 11-octadecenoic acid, methyl ester, and pentadecanoic acid, 14-methyl-, methyl ester which may be responsible for the elicited effects by the extracts of *Carica papaya* seed in this study.

## Declarations

### Author contribution statement

R. Agada, W.A. Usman, S. Shehu and D. Thagariki:: Conceived and designed the experiments; Performed the experiments; Analyzed and interpreted the data; Contributed reagents, materials, analysis tools or data; Wrote the paper.

### Funding statement

This research did not receive any specific grant from funding agencies in the public, commercial, or not-for-profit sectors.

### Competing interest statement

The authors declare no conflict of interest.

### Additional information

No additional information is available for this paper.
